# Analysis of Morphologic and Functional Outcomes in Macular Edema due to Central Retinal Vein Occlusion Treated with Intravitreal Dexamethasone Implant

**DOI:** 10.1155/2018/5604632

**Published:** 2018-09-09

**Authors:** Alfredo Niro, Giancarlo Sborgia, Alessandra Sborgia, Luigi Sborgia, Claudio Furino, Nicola Recchimurzo, Giovanni Alessio

**Affiliations:** Department of Medical Science, Neuroscience and Sense Organs, Eye Clinic, University of Bari “A. Moro”, Bari, Italy

## Abstract

**Purpose:**

To analyze anatomic and functional retinal changes and their correlation after intravitreal dexamethasone implant (DEX implant) in patients with central retinal vein occlusion- (CRVO-) related macular edema (ME) using optical coherence tomography and microperimetry.

**Methods:**

Fifteen treatment-naïve patients with functional impairment due to CRVO-related ME were enrolled in this prospective interventional case series. Main outcomes were best-corrected visual acuity (BCVA), retinal sensitivity (RS), and central retinal thickness (CRT). Secondary outcomes were ellipsoid zone (EZ) status and fixation behaviour. All patients underwent DEX implant and were retreated according to predefined criteria. Data were prospectively recorded at baseline and at month 1, 3, 6, 9, and 12. Correlation between main outcomes was analyzed.

**Results:**

Fifteen eyes of 15 patients (9 men, 6 women; mean age 61.8 ± 10.9 years) were included. BCVA and CRT significantly improved at all follow-up visits, while RS significantly improved at 3, 6, 9, and 12 months. EZ status and fixation behaviour did not change significantly. Baseline CRT had a significant negative correlation with BCVA and RS at different follow-up visits (*r*=−0.52  to −0.63, *p* ≤ 0.04; *r*=−0.52, *p*=0.04; resp.). At all time points, there was not a significant correlation between CRT and BCVA and RS, while RS and BCVA showed a significant correlation, increasing over time (*r*=−0.72  to −0.89; *p* < 0.001).

**Conclusion:**

DEX implant led to a significant morphofunctional improvement. Baseline CRT is predictive of changes of functional outcomes whose correlation increases over time after treatment.

## 1. Introduction

Macular edema (ME) is a common complication of central retinal vein occlusion (CRVO) causing vision loss [1]. The pathogenesis of ME in retinal vein occlusion is not completely understood but may result from a variety of factors, including hydrostatic effects from increased venous pressure, the presence of inflammatory cytokines, the dysregulation of endothelial tight junction proteins [2], or increased amounts of vascular permeability factors, such as vascular endothelial growth factor (VEGF) [3]. Treatment options include intravitreal corticosteroids and antagonists of VEGF (anti-VEGF) [4–12]. The slow-release intravitreal dexamethasone implant 0.7 mg (DEX implant; Ozurdex®, Allergan plc, Dublin, Ireland) has proved very effective improving visual acuity and reducing macular thickness in patients with CRVO-related ME [13, 14]. Best-corrected visual acuity (BCVA) and central retinal thickness (CRT) assessed by optical coherence tomography (OCT), reflecting foveal status, are widely used as efficacy outcomes of DEX implant in clinical trials and clinical practice. However, visual acuity and its relationship with CRT could not be comprehensive in monitoring the morphologic and functional recovery of a larger retinal area beyond the fovea after DEX implant. Retinal sensitivity (RS), assessed by microperimetry, as a reproducible point-to-point measure of macular function was used as a biomarker of functional changes in retinal vein occlusion [15, 16] and recovery after DEX implant [17–22]. However, the correlation between morphologic and functional parameters and their predictive role for recovery after DEX implant over a long follow-up remains unclear. So we analyzed both CRT and visual function parameters, as BCVA and RS, and their correlation in CRVO-related ME patients after DEX implant over 12-months follow-up.

## 2. Methods

This was a prospective interventional case series of intravitreal dexamethasone implant (Ozurdex) in consecutive naive patients with visual impairment and clinical and/or instrumental diagnosis of ME following CRVO within previous three months before enrollment. The main exclusion criteria were a BCVA worse than 1.5 logMAR, previous intravitreal implant or injection, previous vitreoretinal surgery, manifest glaucoma, presence of diabetic macular edema, epiretinal membrane, retinal neovascularization, previous central or branch retinal vein occlusion, retinal dystrophies, severe cataract, and other diseases affecting visual acuity in the affected eye. All subjects underwent DEX implant and were followed at month 1, 3, 6, 9, and 12. All subjects underwent a complete examination including BCVA evaluation, slit-lamp biomicroscopy, dilated fundus examination, intraocular pressure (IOP) measurement, optical coherence tomography (OCT; Cirrus HD-OCT Zeiss, Carl Zeiss Meditec, Germany), and microperimetry (MP1; Navis Software, version 1.7.6; Nidek Technologies, Padova, Italy). Fluorescein angiography was performed at baseline, 4 and 12 months. Primary outcome measures were BCVA, CRT measured by OCT, and RS measured by MP1. BCVA was evaluated using a standardized Early Treatment Diabetic Retinopathy Study (ETDRS) protocol. We used a macular thickness map protocol with a macular cube 512 × 128 combo to measure CRT as the mean thickness in the central 1000-*µ*m diameter area, considering the distance between the internal limiting membrane and outer border of the photoreceptor cells [23]. RS was considered as the mean sensitivity measured across a 45-point grid centered on the central 12 degrees using pattern Macula 12°–0 dB. At each point in the grid, sensitivity was measured for a white stimulus 0.438 in diameter (Goldmann size III) presented for 200 msec against a mesopic background (1.27 cd/m^2^). Threshold at each point was determined by using a 4-2 staircase. When the automatic localization of the foveal area was difficult, that site has been manually located at a distance of two papillary disc temporally and a disc-third lower than the center of the optic disc. The “follow-up” feature of the MP1 was used to perform sensitivity measurements at the same retinal locations across all visits. Secondary outcomes were the integrity of the ellipsoid zone (EZ) at the foveal site revealed by the continuity of hypereflective band of photoreceptor inner/outer segment junction at horizontal OCT high-resolution linear scans (HD 5-lines) and classified as absent or present and fixation stability revealed by plotting the position of each fixation point on Cartesian axes and calculating the percent of the points falling within 2° and 4° circle by using MP-1 software as recommended by Fujii et al. [24]. All patients were retreated from month 4 according to an open pro re nata (PRN) regime (if either a significant decline in the BCVA, as demonstrated by a loss of at least one line, or an increased CRT of ≥100 *µ*m was noticed from the previous visit), provided the patient had not experienced raised IOP above 30 mm Hg following their first injection. Additionally, supplementing sectorial or panretinal laser photocoagulation was considered on the individual retinal perfusion. Ocular and systemic complications were recorded. The treatment described adhered to the tenets of the Declaration of Helsinki. Written informed consent for implant for one eye each and research aim was requested.

### 2.1. Statistical Analysis

Statistical analysis was based on all patients included in the study. No formal sample size calculation was performed. Baseline was defined as the day before implant. A *t*-test was performed on the change from baseline in CRT, BCVA, and RS. The statistical analysis for the evaluation of the correlation between primary outcomes was based on the observed data collected during the 12-month study period and examined by Pearson correlation coefficient. All statistical tests were 2-sided and performed at the *p*0.05 significance level. Data processing, summaries, and analysis were performed using the statistical software package SAS version 9.1 or higher (SAS Institute Inc, Cary, NC).

## 3. Results

**Figure 1:**
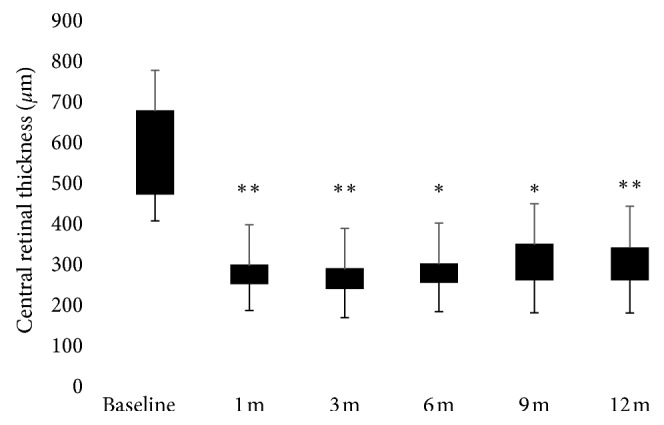
Central retinal thickness (CRT) improvement over time (*µ*m: micronmeters; m: month(s)). ^*∗*^*p*  0.002; ^*∗∗*^*p* < 0.0001.

**Figure 2:**
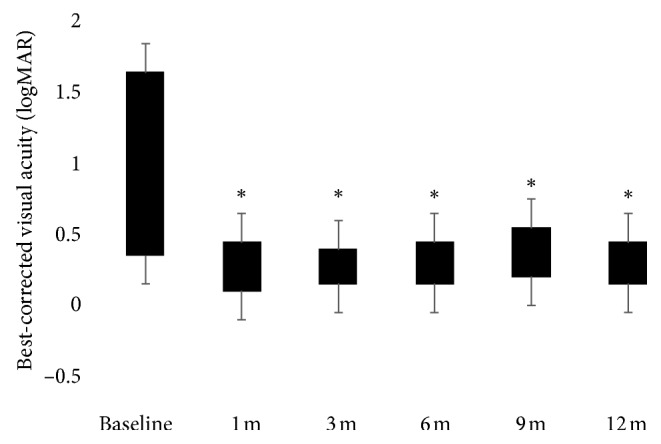
Best-corrected visual acuity improvement over time (logMAR: logarithm of the minimum angle of resolution; m: month(s)). ^*∗*^*p* < 0.001.

**Figure 3:**
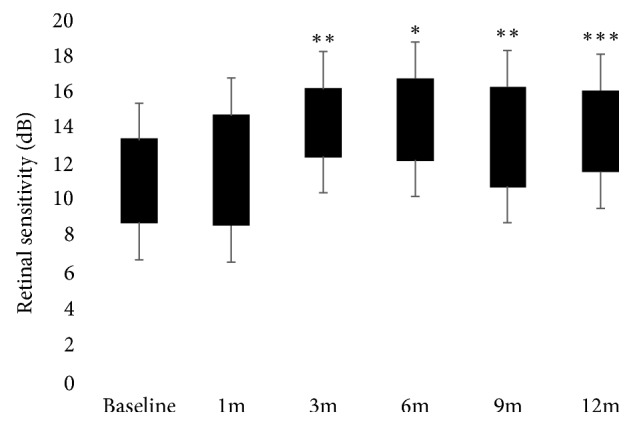
Retinal sensitivity improvement over time (dB: decibels; m: month(s)). ^*∗*^*p* < 0.01; ^*∗∗*^*p* < 0.001; ^*∗∗∗*^*p* < 0.0001.

The baseline characteristics of the study population are summarized in Table 1. Only 15 consecutive patients out of 21 CRVO-related ME patients who underwent dexamethasone slow-release implant were included in this study because 6 patients refused consent for research. Mean age of the 9 men and 6 women included was 61.8 ± 10.9 years (range: 46–80 years). Due to the recurrence of ME, a second DEX implant was performed in two and two eyes at 6 and 9 months, respectively. Six eyes underwent laser treatment during follow-up. The mean CRT significantly decreased from 577 ± 170.2 *μ*m at baseline to 287.7 ± 44.2 *μ*m (*p* < 0.0001) at 1 month, to 271.87 ± 43.8 *μ*m (*p* < 0.0001) at 3 months, to 316.3 ± 123 *μ*m (*p*= 0.0002) at 6 months, to 318.4 ± 90.2 *μ*m (*p*= 0.0002) at 9 months, and to 294.1 ± 60.6 *μ*m (*p* < 0.0001) at 12 months (Figure 1). Respect to baseline CRT, the largest mean thickness reductions were observed at 3 (289.3 ± 126.5 *µ*m) and 12 months (282.8 ± 109.6 *µ*m) after implant. At 6 and 9 months, an increase of 44.73 ± 120.7 *µ*m and 47.33 ± 98.06 *µ*m of the mean CRT, respectively, respect to the earliest 3 months, occurred. The analysis of the EZ integrity at the foveal site was realized from the first follow-up visit at 1 month because at baseline large intraretinal cystic spaces masked the reflectivity of the outer layers at OCT scans. Two patients had a discontinuity of the reflectivity of the EZ at months 1 and 12 after implant. OCT scans highlighted occurrence of a discontinuity in the EZ in only one patient at last follow-up (Table 2). At baseline, OCT scans revealed subretinal fluid in 5 patients. The mean BCVA significantly improved from 0.76 ± 0.54 logMAR at baseline to 0.37 ± 0.33 logMAR at 1 month (*p* < 0.001), 0.34 ± 0.33 logMAR at 3 months (*p* < 0.001), 0.37 ± 0.34 logMAR at 6 months (*p* < 0.001), 0.38 ± 0.31 logMAR at 9 months (*p* < 0.001), and 0.35 ± 0.32 logMAR at 12 months (*p* < 0.001) (Figure 2). Respect to baseline BCVA, the largest mean visual improvements of 0.42 ± 0.38 logMAR and 0.41 ± 0.36 logMAR were seen at 3 and 12 months, respectively. At 6 and 9 months, a mean visual impairment of 0.05 ± 0.08 LogMAR and 0.12 ± 0.10 logMAR, respectively, respect to the earliest 3 months, occurred. The mean RS improved from 10.39 ± 5.03 dB at baseline to 11.86 ± 5.91 dB at 1 month (*p* > 0.05), 13.03 ± 6.09 dB at 3 months (*p* < 0.001), 12.92 ± 7.1 dB at 6 months (*p* < 0.01), 13.03 ± 5.86 dB at 9 months (*p* < 0.001), and 13.14 ± 5.6 dB at 12 months (*p* < 0.0001). (Figure 3) Respect to baseline sensitivity, the largest mean sensitivity improvements of 2.77 ± 2.28 dB and 2.89 ± 2.29 dB were seen at 3 and 12 months, respectively. At 6 months, a mean sensitivity impairment of 0.53 ± 0.4 dB from earliest 3 months occurred. In the four eyes retreated, the second implant gave a mean CRT (170 ± 93.5 *µ*m) and functional improvement (0.2 ± 0.3 LogMAR; 0.5 ± 1.1 dB). In regard to the fixation behaviour, comparing baseline and last follow-up data, three patients revealed a mild improvement (unstable to relatively stable) while two patients suffered a mild impairment (stable to relatively unstable) in the fixation stability within central 4° (Table 2). The baseline CRT was significantly correlated with BCVA at months 1, 3, 6, and 12 (*r*=−0.52  to − 0.63, *p* ≤ 0.04) (Table 3). At each follow-up visit, BCVA and CRT showed a weak correlation (*r*=−0.07  to − 0.26) never statistically significant (*p* > 0.05). The baseline CRT was significantly correlated with RS at 9 months (*r*=−0.52*p*=0.04) after treatment (Table 3). At each follow-up visit RS and CRT showed a weak correlation (*r*=−0.40  to − 0.46) never statistically significant (*p* > 0.05). At each time point, BCVA and RS showed a strong negative correlation (*r*=−0.72  to − 0.96, *p* < 0.01), increasing over time (Table 4). At 6 and 9 months after a single implant, OCT scans revealed vitreomacular interfaces disorders (VID), not present at baseline, in a total of 6 patients. In one patient, a significant cataract progression occurred after two implants, so he underwent surgery during follow-up. In two patients, IOP elevation (<30 mmHg) was successfully managed with antihypertensive topical therapy, suspended after 1 month from implant.

## 4. Discussion

Ozurdex may offer patients with CRVO-related ME a long-lasting relief from visual symptoms and edema with a limited number of treatments and follow-up visits [25–27]. In our study, DEX implant significantly improved primary outcomes, such as CRT and BCVA at all follow-up visits. By analyzing our results and comparing with results of GENEVA, OCTOME (Report 1), and SOLO studies, albeit with a nonoverlapping follow-up, we found a similar trend in morphologic and functional recovery. A significant mean visual improvement (0.42 ± 0.38 logMAR) and CRT reduction (289.3 ± 126.5 *µ*m) occurred over early three months, followed by a mild functional impairment and increase in mean CRT at the sixth and ninth month compared to previous time points, due to edema recurrence and/or VID or cataract occurrence, and a new mean morphofunctional recovery, lower than the previous, after the second implant, as reported by the literature [13, 22]. With regard to RS, we found the same trend with an early improvement of 2.77 ± 2.28 dB at 3 months, followed by a mild impairment at the sixth and ninth month compared to previous visits. At 12 months, a new mean sensitivity improvement (2.89 ± 2.29 dB) was recorded. The ME recurrence at the sixth and ninth month justified an additional implant in overall 4 (26.6 %) patients, really lower than 50% of patient retreated in GENEVA Study. All these results might suggest a correlation between macular sensitivity, visual acuity, and retinal thickness. In this study, we evaluated the correlation between morphologic and functional parameters as outcomes of intravitreal DEX implant administered on a PRN regime for CRVO-related ME. Noma et al. found that BCVA was significantly correlated with macular thickness in eyes with CRVO-related ME [15]. Querques et al. reported that macular thickness was positively correlated with BCVA at months 1 and 3 after DEX implant [18]. Bulut et al. observed a moderate correlation between macular thickness and BCVA only at 3 months after DEX implant. No correlation was detected between BCVA and macular thickness at baseline and 6 months [28]. We found that baseline CRT had a significant negative correlation with visual acuity at different follow-up visits (at months 1, 3, 6, and 12) after implant, but conversely, CRT had a weak correlation with simultaneous visual acuity at each time points. That data suggest that baseline CRT could be considered a predictive factor for visual acuity changes after treatment, but the weak correlation at each time point could reveal that anatomical degenerative changes caused by vein occlusion influence functional parameters over time, regardless of macular thickness. Furthermore, baseline CRT showed no significant relationship with BCVA at 9 months. CRT could have only a time-limited predictive role and not have an explanatory role on visual acuity recovery over follow-up, probably due to later neuroretinal modifications occurring beyond the period of efficacy of DEX implant. So, it should not be the only morphologic finding recorded. In the same way, the functional changes cannot be completely assessed by visual acuity. Visual acuity primarily reflects foveal function, but retinal sensitivity studied by using microperimetry, creating a map of macular sensitivity, records the central and paracentral retinal function that visual acuity is not able to assess. OCTOME study (Report 1) reported a sensitivity improvement of 4 dB in a population affected by different retinal vascular diseases at 6 months after DEX implant [22]. Several papers reported a mean sensitivity improvement ranging from 1.1 dB to 3.9 dB over a variable follow-up period (3 to 12 months) after DEX implant [17–21]. In our study, including only patients with CRVO-related ME over a 12-month follow-up, a RS mean recovery of 2.75 dB was recorded with a slower trend than visual acuity. Noma et al. reported a significant correlation between baseline CRT and RS [15]. Querques et al. found a negative significant correlation between macular thickness and retinal sensitivity over 3 months after DEX implant [17, 18]. We found a negative significant correlation between baseline CRT and RS only at nine months after implant. This finding supports the complex interaction between morphologic and functional parameters. Not only the retinal thickness but also the compartmentalization of edema involving neurosensory layers and subretinal space was found to contribute differently to the reduction and recovery of visual function [29, 30]. A relationship between ganglion cell layer thinning and a decrease in local visual field sensitivity and visual acuity has been found in several studies [31]. Inner retinal layers displacement, dysfunction, and loss affecting a greater area than foveal site due to fluid accumulation may explain decreased retinal sensitivity out of the fovea [32]. The sensitivity recovery is influenced by the reconstitution of neuroretinal architecture after retinal edema resolution. But these changes could occur over a long time justifying the slow recovery trend of retinal sensitivity. Furthermore, the reduction of retinal thickness does not imply an immediate functional recovery. With worsening ischemia, the increased interstitial pressure causing compromised perfusion and the tissue reperfusion might lead to irreversible damage of the neuroretina influencing functional recovery, so BCVA and RS, differently, can remain poor even though the ME is resolved. Previous papers revealed a reduction of foveal and parafoveal retinal superficial and deep vascular density and choriocapillaris density in eyes with vein occlusion-related ME [33–35]. Although Mastropasqua et al. found that retinal vessel plexi density did not change significantly after DEX implant and there was no correlation between vessel density and functional parameters such as visual acuity and retinal sensitivity [36], it has been demonstrated that intravitreal steroid, in eyes with macular edema due to retinal vein occlusion or diabetic retinopathy, causes a reduction of vessel diameter probably due to the blockage of VEGF [37, 38]. Vessel occlusion may lead to functional damage of photoreceptor cells that influence visual acuity and retinal sensitivity. This effect on photoreceptor cells could explain the significant negative correlation between visual acuity and retinal sensitivity and its progressive increase over follow-up. In our study, five patients had subretinal fluid at baseline, and three of those showed an EZ discontinuity at last follow-up. These three patients had worst baseline BCVA (range, 1.3–1.5 logMAR) and RS (range, 1.2–2 dB) and worst final visual outcomes (range, 0.7–1 logMAR; 5.8–2 dB). SCORE study (Report 13) highlighted the correlation between functional parameters and outer retinal layer integrity [39]. In retinal vein occlusion [40, 41] or diabetic macular edema [42], as well as in age-related macular degeneration [43], a significant association between changes of EZ and BCVA was reported, on the other hand, they did not find a statistically significant correlation between photoreceptor layer feature and CRT. OMAR study revealed that visual acuity was better for BRVO, although CRT showed no significant difference between central and branch vein occlusion groups [44]. Altunel et al. found that outer nuclear layer thickness and photoreceptor layer thickness were more closely related to visual acuity improvement than CRT decrement [45]. The CRT before the treatment and the presence of an intact EZ at the time of the ME resolution were significantly correlated with the BCVA and RS at 6 months after anti-VEGF therapy for retinal vein occlusion [46]. RS has been shown to be significantly correlated not only with the BCVA but also with morphologic findings, such as EZ status, and it can thus be used to monitor the effectiveness of treatments [47–49] CRT reflects the status of the edema not revealing outer retina integrity at the foveal site. Histologic studies have shown that severe ME leads to photoreceptor dysfunction and photoreceptor cell loss [50]. However, OCT scans show neuroretinal swelling due to edema, masking the EZ that could be preserved or interrupted. We evaluated outer retinal layers at 1 month after treatment because the pretreatment status of the EZ could be masked by the edema. A significant visual gain was achieved only in eyes showing preserved outer retinal layers at baseline regardless of the CRVO types, whereas eyes with absent layers at baseline were unable to attain any visual improvement [51]. With regard to the fixation behaviour, it did not show significant changes from baseline to 12-month follow-up. It was stable or partially stable in the majority of patients. However, fixation stability was not related to visual acuity, and the compensation of fixation instability does not improve visual acuity in patients with macular disease [52]. In the present study, multivariate analysis was not performed because the statistical model was unsuitable due to the small number of patients. This study also had some other limitations: the eyes were not categorized according to ischemic or nonischemic feature of CRVO; study visits were not scheduled between months 1, 3, and 6, therefore not revealing the exact time point of drug peak efficacy, its corresponding duration, and possible need for early retreatment. It must be said for completeness of analysis which is inherent connotation of each psychophysical test and as such also the microperimetry, the measurement error, or intrinsic variability. Factors acting on test variability are related to patients' compliance and their anatomical and functional condition and to the examiner and/or instrument used. An important patient-related factor influencing test execution is the “learning factor” which can justify a certain degree of improvement during follow-up. With regards to the instrument, it should be mentioned the eye-tracker system, not able to ensure the same accuracy of analysis between the posterior pole and peripheral retina; the “point to point” overlapping error (0.5° to 2°) when “follow-up” program is used; the “4-2 strategy” which can extend the duration of the test; the “ceiling effect” of MP-1, meaning the tendency to accumulate responses at the highest limit of the sensitivity threshold; the size of the given stimulus (Goldmann III, 4 mm^2^ area, 26 min diameter of arc, or 0.4°) that, because of “spatial summation,” involves more photoreceptors which converge centrally on a single ganglion cell; the wider extension of tested area (central 20°) respect to central 4° and 8° which would result in a more reliable parameter for spatial localization of functional defect. Further investigations will be needed to clarify the relations between macular function and morphology in CRVO patients with macular edema. In conclusion, beyond the efficacy of DEX implant, our analysis suggests that baseline CRT might have a prognostic role on functional changes and that mutual behaviour of functional parameters over time might be related to the restoration of the integrity of photoreceptor cells after macular edema resolution. So, comprehensive evaluation of both visual acuity and macular sensitivity might be important when assessing retinal function in CRVO patients with macular edema before and after DEX implant and their correlation could reveal the added value of microperimetry as a diagnostic tool.

## Figures and Tables

**Table 1:** Patient characteristics.

Patient, *n*	15
Age (years ± SD)	61.8 ± 10.9
Sex (men/women), *n*	9/6
Retreated eyes, *n*	4/15
Eyes underwent laser, *n*	6/15

SD, standard deviation.

**Table 2:** Secondary outcomes.

EZ status at 1 month (absent/present), *n*	2/13	
EZ status at 12 months (absent/present), *n*	3/12	
Fixation stability within 4 degrees, *n*	Baseline	12 months

Stable	11/15	8/15
Relatively stable	—	2/15
Relatively unstable	—	4/15
unstable	4/15	1/15

EZ: ellipsoid zone.

**Table 3:** Correlation between baseline CRT and main functional outcomes.

	Baseline CRT				
	1 month	3 months	6 months	9 months	12 months
BCVA	*r*=−0.55	*r*=−0.63	*r*=−0.55	*r*=−0.40	*r*=−0.52
	*p*=**0.03**	*p*=**0.01**	*p*=**0.03**	*p*=0.13	*p*=**0.04**

RS	*r*=−0.45	*r*=−0.46	*r*=−0.45	*r*=−0.52	*r*=−0.40
	*p*=0.09	*p*=0.07	*p*=0.09	*p*=**0.04**	*p*=0.13

*Note*. *p* values indicate statistical significance. BCVA: best-corrected visual acuity; RS: retinal sensitivity; CRT: central retinal thickness.

**Table 4:** Correlation between BCVA and RS.

	BCVA					
	Baseline	1 month	3 months	6 months	9 months	12 months
RS	*r*=−0.72	*r*=−0.82	*r*=−0.91	*r*=−0.93	*r*=−0.96	*r*=−0.90
	*p*=**0.002**	*p*=**0.0001**	*p* < **0.00001**	*p* < **0.00001**	*p* < **0.00001**	*p* < **0.00001**

BCVA: best-corrected visual acuity; RS: retinal sensitivity.

## Data Availability

The data used to support the findings of this study are available from the corresponding author upon request.
